# RETRACTION: Laparoscopic vs. Open Surgery: A Comparative Analysis of Wound Infection Rates and Recovery Outcomes

**DOI:** 10.1111/iwj.70470

**Published:** 2025-04-02

## Abstract

**RETRACTION:** ShiZ. , “Laparoscopic vs. Open Surgery: A Comparative Analysis of Wound Infection Rates and Recovery Outcomes,” International Wound Journal 21, no. 3 (2024): e14474, 10.1111/iwj.14474.37905679 PMC10898397

The above article, published online on 31 October 2023, in Wiley Online Library (http://onlinelibrary.wiley.com/), has been retracted by agreement between the journal Editor in Chief, Professor Keith Harding; and John Wiley & Sons Ltd. Following an investigation by the publisher, all parties have concluded that this article was accepted solely on the basis of a compromised peer review process. The editors have therefore decided to retract the article. The authors did not respond to our notice regarding the retraction.

**RETRACTION:** Z. Shi , “Laparoscopic vs. Open Surgery: A Comparative Analysis of Wound Infection Rates and Recovery Outcomes,” International Wound Journal 21, no. 3 (2024): e14474, 10.1111/iwj.14474.37905679 PMC10898397

The above article, published online on 31 October 2023, in Wiley Online Library (http://onlinelibrary.wiley.com/), has been retracted by agreement between the journal Editor in Chief, Professor Keith Harding; and John Wiley & Sons Ltd. Following an investigation by the publisher, all parties have concluded that this article was accepted solely on the basis of a compromised peer review process. The editors have therefore decided to retract the article. The authors did not respond to our notice regarding the retraction.

